# Laser-Induced Linear-Field Particle Acceleration in Free Space

**DOI:** 10.1038/s41598-017-11547-9

**Published:** 2017-09-11

**Authors:** Liang Jie Wong, Kyung-Han Hong, Sergio Carbajo, Arya Fallahi, Philippe Piot, Marin Soljačić, John D. Joannopoulos, Franz X. Kärtner, Ido Kaminer

**Affiliations:** 1 Department of Mathematics, Massachusetts Institute of Technology, 77 Massachusetts Avenue, Cambridge, 02139 Massachusetts, USA; 2 Department of Physics, Massachusetts Institute of Technology, 77 Massachusetts Avenue, Cambridge, 02139 Massachusetts, USA; 3 Singapore Institute of Manufacturing Technology, 2 Fusionopolis Way, Innovis, 138634 Singapore; 4 Department of Electrical Engineering and Computer Science and Research Laboratory of Electronics, Massachusetts Institute of Technology, 77 Massachusetts Avenue, Cambridge, 02139 Massachusetts, USA; 5 Hamburg Center for Ultrafast Imaging, Luruper Chaussee 149, 22607 Hamburg, Germany; 6 Center for Free-Electron Laser Science, Deutsches Elektronen-Synchrotron, Notkestraße 85, D-22607 Hamburg, Germany; 7 Department of Physics and Northern Illinois Center for Accelerator & Detector Development, Northern Illinois University, DeKalb, IL 60115 USA; 8 Fermi National Accelerator Laboratory, Batavia, IL 60510 USA; 9 Department of Electrical Engineering, Technion−Israel Institute of Technology, 32000 Haifa, Israel; 10 Stanford University and SLAC National Accelerator Laboratory, 2575 Sand Hill Road, Menlo Park, CA 94025 USA

## Abstract

Linear-field particle acceleration in free space (which is distinct from geometries like the linac that requires components in the vicinity of the particle) has been studied for over 20 years, and its ability to eventually produce high-quality, high energy multi-particle bunches has remained a subject of great interest. Arguments can certainly be made that linear-field particle acceleration in free space is very doubtful given that first-order electron-photon interactions are forbidden in free space. Nevertheless, we chose to develop an accurate and truly predictive theoretical formalism to explore this remote possibility when intense, few-cycle electromagnetic pulses are used in a computational experiment. The formalism includes exact treatment of Maxwell’s equations and exact treatment of the interaction among the multiple individual particles at near and far field. Several surprising results emerge. We find that electrons interacting with intense laser pulses in free space are capable of gaining substantial amounts of energy that scale linearly with the field amplitude. For example, 30 keV electrons (2.5% energy spread) are accelerated to 61 MeV (0.5% spread) and to 205 MeV (0.25% spread) using 250 mJ and 2.5 J lasers respectively. These findings carry important implications for our understanding of ultrafast electron-photon interactions in strong fields.

## Introduction

The prospect of realizing high-gradient linear particle accelerators on small laboratory or portable scales has stimulated immense interest in laser-driven electron acceleration^1–12^. At the heart of these schemes lies the critical Lawson-Woodward theorem^13–15^, which states that any laser-driven *linear-field* acceleration of charged particles (defined by net energy gain that scales linearly with field amplitude) cannot occur in free space. Net energy gain in free space must involve interactions beyond first-order in perturbation theory (i.e., higher order diagrams in quantum electrodynamics), since first-order interactions between electrons and photons are forbidden in free space^16^. Thus, laser acceleration schemes typically employ assisting media like plasma^1, 10, 11^ or nearby dielectric structures^9, 17, 18^ to facilitate a first-order interaction. The question of whether one can achieve net linear-field particle acceleration without assisting media has been studied for over 20 years, with various proposals for such schemes studied using approximate treatments. Serious concerns, however, have been raised regarding the validity of these approximate treatments^7, 19, 20^. Here, we present exact, many-body, ab-initio simulations showing that the Lawson-Woodward theorem can be bypassed to achieve monoenergetic acceleration of a multi-electron bunch in *free space*, using the longitudinal field of an ultrafast laser pulse. Importantly, the multi-electron nature of the problem necessitates a treatment in which inter-electron interactions are taken into account, which our model provides exactly. As examples, 30 keV electrons (2.5% energy spread) are accelerated to 7.7 MeV (2.5% spread) and 205 MeV (0.25% spread) using 25 mJ and 2.5 J lasers respectively. We find that despite the interaction occurring in free space, where first-order interactions are forbidden, the energy gain can scale *linearly* in the laser field amplitude; this has important implications for our understanding of ultrafast electron-photon interactions in strong fields. Note that although we use 30 keV electrons as a case study here, exact simulations (Supplementary Information Section 1) show that substantial net acceleration can also be obtained for highly relativistic electrons. Specifically, substantial net linear-field acceleration is possible if the laser pulse is powerful enough to take the particle between relativistic and non-relativistic regimes, in the initial particle’s rest frame. These findings suggest new, exciting opportunities in the development of *compact*, high-quality, ultra-relativistic electron sources that avoid conventional limits imposed by material breakdown or structural constraints.

Interest in the possibility of laser-driven electron acceleration began as early as the 1970s and grew rapidly in the decades that followed, fueled by the invention of chirped pulse amplification in the 1980s^21^ and a steady trend toward laser pulses of higher energies and intensities^2^. The proposal of laser-plasma acceleration^1^ in 1979, for instance, was followed by a period of active research culminating in direct experimental demonstrations of the concept in the 1990s (e.g. ref. 22). However, it was not until 2004 that a regime for *monoenergetic* relativistic acceleration (e.g., 80–90 MeV electrons with few-percent energy spreads using 0.5 J lasers) was discovered^23–25^, allowing the scheme to generate the large current beams with low energy spread necessary for many high-energy electron beam applications. More recently, demonstrations of laser-plasma acceleration have produced 200–600 MeV electron bunches containing several tens of pC of charge using a 6 J laser^26^, as well as MeV electrons using mJ levels of laser energy^27, 28^. The broad spectrum of proposed laser-based acceleration schemes also includes inverse Čerenkov acceleration^29^, inverse free-electron lasers^30, 31^, particle acceleration in an active medium (a.k.a., the PASER)^32^, wakefield acceleration^1, 22^, as well as dielectric laser accelerators^12^. All of these schemes, however, use some form of media or nearby material structures, imposing limitations in intensity and current due to practical considerations like material breakdown and damage or laser-plasma instabilities.

Because these limits do not exist in *free space*, laser-driven acceleration in free space has the potential to take full advantage of the extremely high acceleration gradients of focused, intense laser pulses, which are becoming more powerful every year following a Moore-like law. *Linear-field* acceleration schemes of charged particles in free space are especially noteworthy as they have advantages over non-linear (e.g. refs 33 and 34) acceleration schemes in being less subject to transverse fields that increase the radial spread of electrons through mechanisms like ponderomotive scattering^35^, and to radiation losses^36^. Due to the Lawson-Woodward theorem^13–15^, however, one would expect *functional* linear-field acceleration – involving the mono-energetic acceleration of multiple interacting charged particles – to be impossible unless physical media or nearby material structures are present. More recently, several studies have explored other scenarios where multi-electron beams are produced via linear-field acceleration: when the electron dynamics is started or stopped in the midst of the accelerating laser pulse, as in the injection of electrons into the middle of the pulse^3, 34, 37–41^, or when the electrons are sampled before they have left the influence of the pulse^42^. These studies suggest the notion that free space must be broken in some way to enable linear-field acceleration of charged particles.

Nevertheless, there have been early indications^43–47^ that substantial linear-field acceleration in free space is possible for a single, on-axis particle – see Supplementary Information (SI) Section S1 for more discussion. Despite these indications, however, it is not clear whether an actual electron pulse composed of multiple electrons can be accelerated in a stable and controlled way. Instead, one might expect that the electron pulse would acquire a large energy variance (e.g., due to inter-electron repulsion), resulting in a distribution of accelerated and decelerated electrons such that the average net acceleration is negligible. This has never been rigorously tested since there exists no study on laser-driven linear-field acceleration that takes into account the inter-particle interactions of a multi-electron bunch, nor the radiation reaction that ensues when an electron radiates as a result of its motion. Importantly, it was also suspected that any predicted linear-field acceleration (e.g. ref. 43) was an artifact of certain approximations, such as the paraxial approximation^7, 19^. The importance of a non-paraxial model has been underscored by previous studies predicting vastly different electron dynamics induced by a non-paraxial wavepacket versus its paraxial counterpart^19, 20^, leading to adoption of non-paraxial descriptions to model different laser-electron interaction phenomena^37, 41, 48, 49^. However, a complete multi-electron and non-paraxial description of linear-field acceleration in free space – where near- and far-field inter-particle interaction, and even radiation reaction, may play a significant role – has not been attempted yet. For the above reasons, the possibility of linear-field acceleration in free space has remained an open subject of great interest.

Examining the possibility of linear-field particle acceleration has an importance that goes far beyond the potential usefulness of such a scheme for practical particle acceleration, as it directly touches a key point in light-matter interactions. One does not expect linear-field particle acceleration to be possible for the following fundamental reason: first-order electron-photon interactions are forbidden by energy and momentum conservation; therefore, any acceleration scheme in free-space must include interactions of second-order (or higher) in perturbation theory. However, one would expect a higher-order interaction to result in an energy gain proportional to a higher power of the field amplitude^16^. Confirming that linear-field particle acceleration is possible implies the existence of higher-order interactions that lead to an energy gain that, counter-intuitively, scales linearly with field amplitude. This reveals an intriguing property of high-order processes in electrodynamical interactions and demonstrates the ability of ultrashort, intense fields to excite non-perturbative physics that exhibit very different scaling laws compared to the perturbative scenario.

To investigate these fundamental issues, we develop an exact, multi-particle electrodynamics simulation tool in which laser-driven linear-field acceleration is treated *without any approximations*. In particular, the near-field and far-field interactions of the multiple particles are treated exactly (see SI Section S2). This provides us with new predictive powers that allow us to explore *monoenergetic* net acceleration of a multi-electron bunch with a radially-polarized laser pulse, and to demonstrate using a rigorous theoretical formalism that functional linear-field acceleration is possible in free space. Our findings suggest that high-gradient linear-field acceleration of electron pulses containing a large number of particles can be achieved with an energy spread comparable to or even smaller than current state-of-the-art acceleration techniques, in spite of the unavoidable inter-particle repulsion between electrons. Our scheme only requires engineering the spatiotemporal structure of light in unbounded free space, avoiding the use of media (e.g., gas or plasma), nearby material boundaries, or static fields. Our findings thus constitute the design of the first free-space linear-field acceleration scheme for realistic multi-electron bunches.

**Figure 1:**
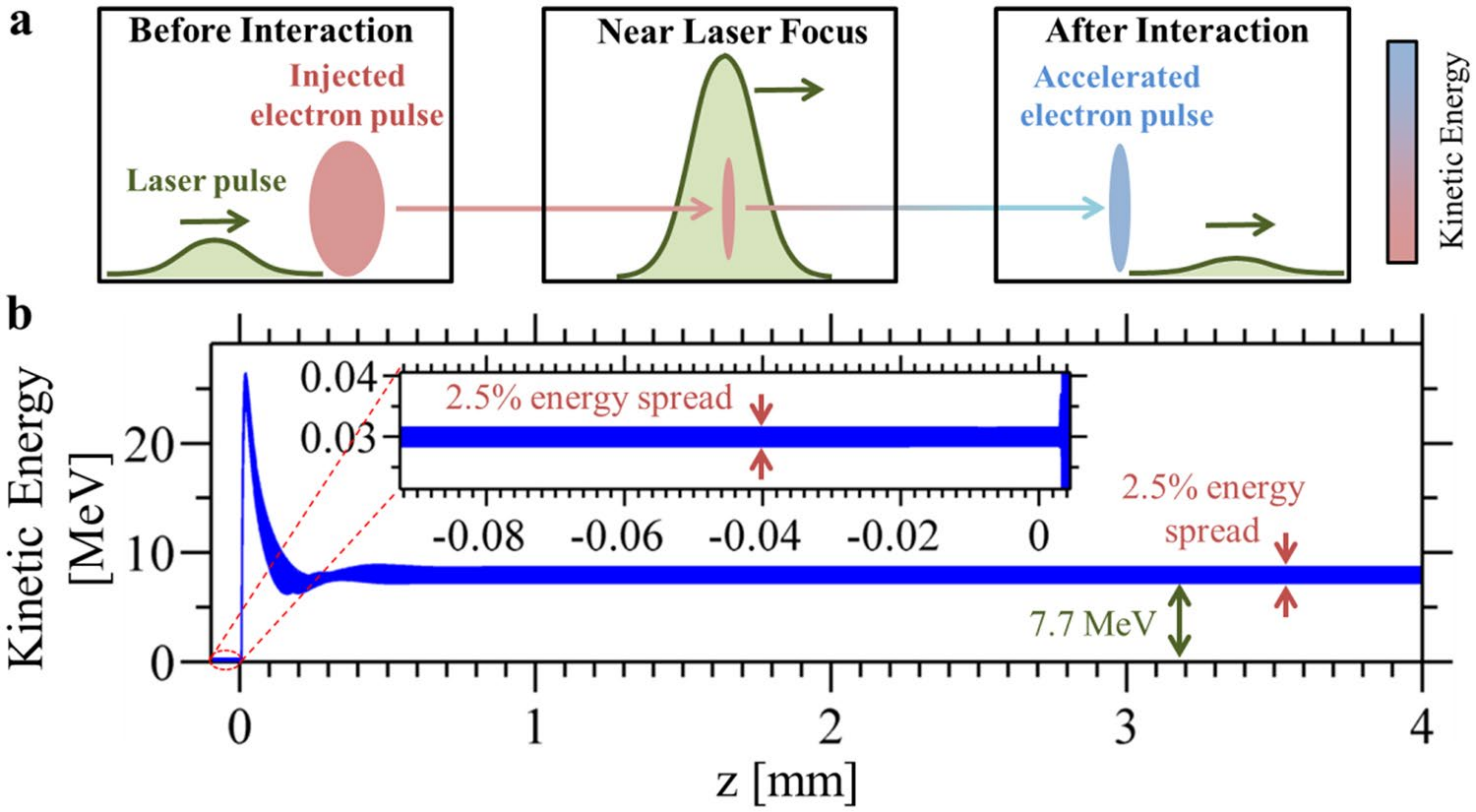
Linear-field electron acceleration in unbounded free space by an ultrafast radially-polarized laser pulse, a process illustrated schematically in (**a**). As an example, (**b**) shows the net acceleration to a final energy of 7.7 MeV of a 30 keV electron pulse with charge −0.2 fC, by a 25 mJ, 3 fs laser pulse of wavelength 0.8 μm focused to a waist radius of 1.6 μm. The initial electrons are randomly distributed in a sphere of diameter 1 μm. More details of the interaction in (**b**) are given in Fig. 2.

Figure 1 shows the acceleration of 30 keV electrons (2.5% spread) to 7.7 MeV (2.5% energy spread) with a 25 mJ pulse. The scheme we study uses the ultrafast radially-polarized laser pulse^50, 51^, an attractive candidate for electron acceleration due to the ability of its transverse fields to confine electrons to the axis exactly where the longitudinal electric field peaks and linear-field particle acceleration is most effective. We use a carrier wavelength of 0.8 μm, full-width-at-half-maximum (FWHM) pulse duration 3 fs (6 fs is studied in SI Section 6), and waist radii (second irradiance moment at focal plane) ranging from *w*_0_ = 0.8 μm to 5.0 μm. It has been shown that such tightly-focused radially-polarized laser beams can be created in practice using parabolic mirrors of high numerical aperture^52^, assisted by wavefront correction with a deformable mirror^53^. Electrons can be injected into the focused region through a small hole on the parabolic mirror. Very recently, millijoule-level, few-cycle pulsed radially-polarized laser beams capable of reaching intensities above 10^19^ W/cm^2^ with kilohertz repetition rates have been experimentally demonstrated^51^. We are also able to verify that the net energy transfer owes itself entirely to the longitudinal electric field component: repeating our simulations under the exact same conditions except for an artificially-zeroed longitudinal electric field component yields negligible electron acceleration.

**Figure 2:**
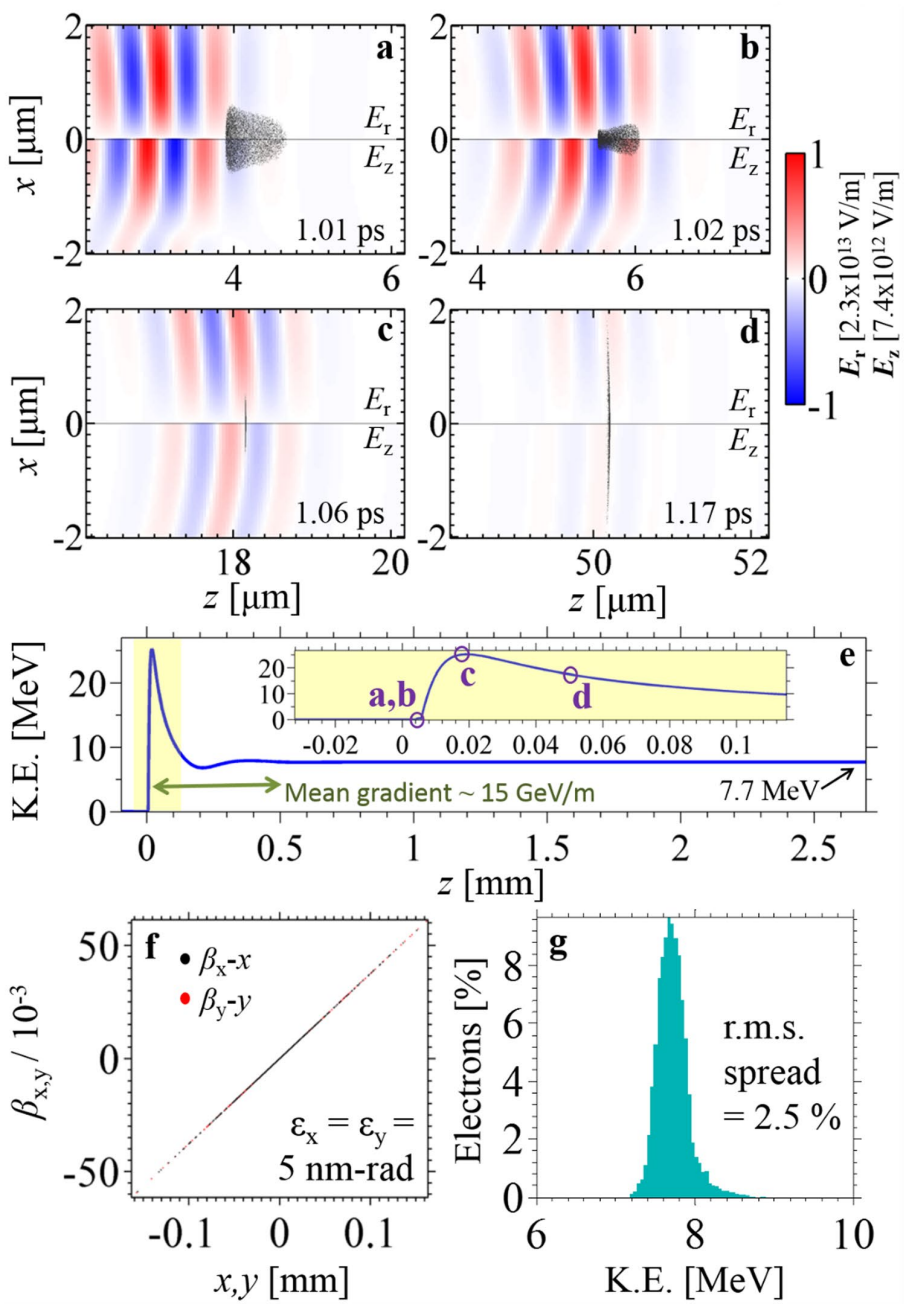
Monoenergetic, relativistic electrons from laser-driven linear-field acceleration in free space. (**a**–**d**) depict the behavior of the optical and electron pulses at various times during the laser-electron interaction. These instants are marked with circles in (**e**), which show the evolution of the electron pulse’s mean kinetic energy as a function of distance (laser focus at *z* = 0). The final (**f**) normalized trace-space emittance and (**g**) energy distribution describe a relativistic, high-quality and quasi-monoenergetic electron pulse. The laser and electron pulse parameters from Fig. 1 were used here. Although the electron pulse eventually acquires a relatively large transverse size (see (**f**)), its low trace-space emittance implies that it is readily re-compressed with appropriate focusing elements (e.g., magnetic solenoid).

Figure 2(a)–(d) capture the laser-driven linear-field acceleration process at various instants (see the Supplementary Video for an animation). At initial time *t* = 0, the injected electrons travel in the complete absence of electromagnetic fields. At $t\approx 1\,{\rm{ps}}$, the optical pulse overtakes the focused electron pulse close to the laser beam focus *z* = 0 (Fig. 2(a)), where the superluminal optical phase velocity causes electrons to slip rapidly through accelerating and decelerating cycles (Fig. 2(b)). As the laser beam diverges, the superluminal phase velocity decreases towards the speed of light c even as the electrons, caught in an accelerating cycle, accelerate towards c, leading to a period of sustained acceleration. At some point, the electrons slip into a decelerating cycle and start losing energy (Fig. 2(c)). Due to the optical beam divergence and finite pulse duration, however, the electrons can still retain a substantial amount of the energy gained after escaping the influence of the optical pulse. The final electron pulse of −0.2 fC, which travels once more in field-free vacuum (Fig. 1a right panel), is quasi-monoenergetic with a mean energy of 7.7 MeV, an energy spread of 2.5% and normalized trace-space emittances^54^ of 5 nm-rad (Fig. 2(d)–(f)). Note that at 7.7 MeV, this corresponds to an unnormalized trace-space emittance of about 0.31 nm-rad, smaller than the initial trace-space emittance of 1 nm-rad. The initial electron bunch is focused to a diameter of 1 μm in each dimension (10 fs pulse duration).

**Figure 3:**
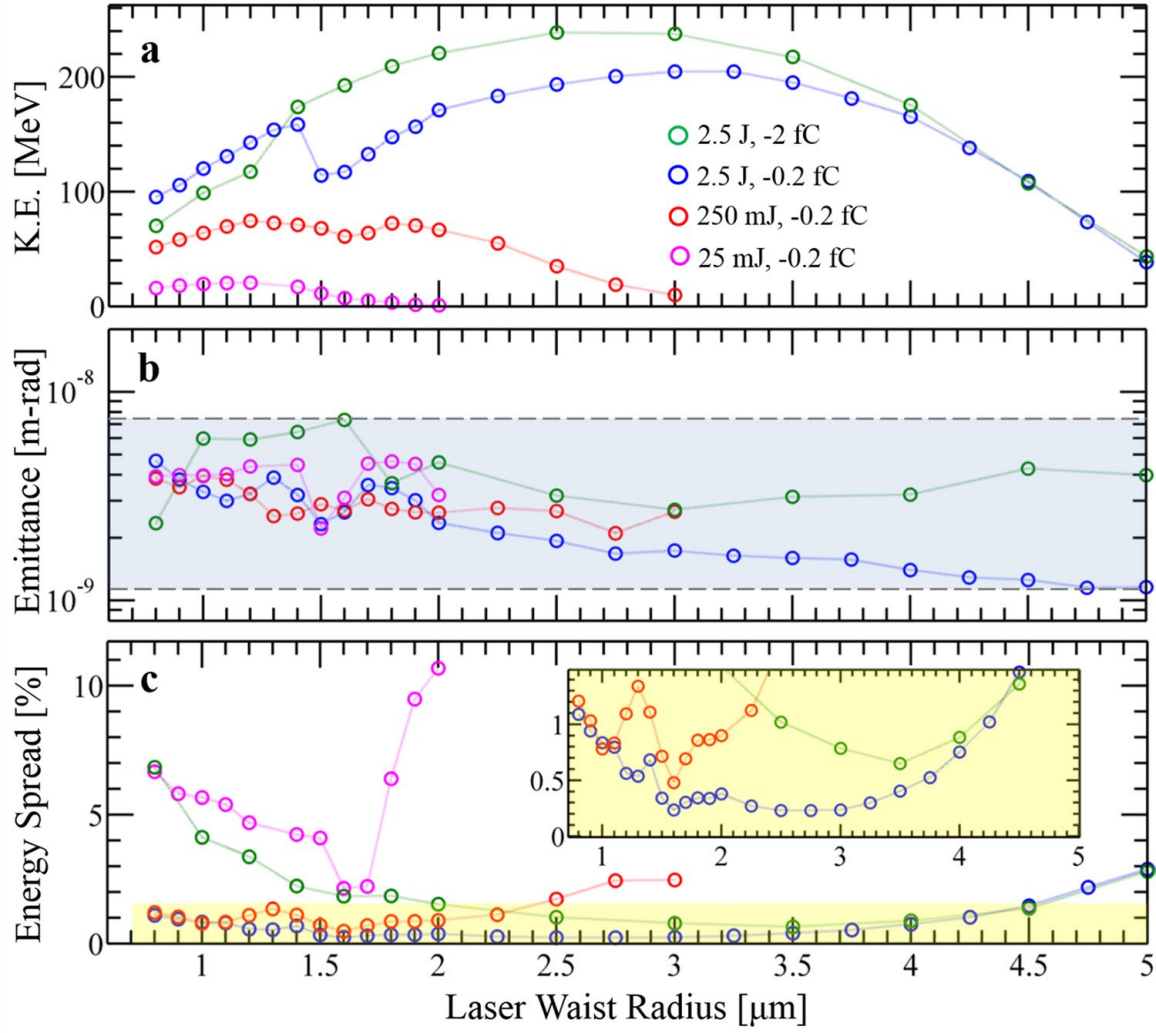
Characteristics of the accelerated electron pulse, showing the insensitivity of laser-driven linear-field acceleration in free space to a wide range of parameter choices. The final (**a**) mean kinetic energy, (**b**) normalized trace-space emittance, and (**c**) energy spread are shown as a function of laser waist radius for various values of laser pulse energy and electron pulse charge. The shaded region between the dashed lines in (**b**) is to highlight the fact that for a wide range of parameters, the final emittance falls in the nm-rad range. We obtain each point by optimizing over the optical carrier phase and the relative displacement between electron and laser focal positions based on our FOM. (Dotted lines are included only as a visual guide).

The generation of initial sub-relativistic electron pulses of few-fC charge, sub-wavelength transverse dimensions and few-femtosecond durations is known to be achievable, e.g., by ionizing a low-density gas with a laser beam^37, 41^, all-optical compression techniques^55, 56^ or photoemission from sharp metal tips^57, 58^. The modest amount of charge considered in our scheme may be scaled up by employing a high-repetition-rate electron source and an external cavity to recycle the laser pulse, leading to average currents as high as 2 μA for a 1 GHz repetition rate. Relaxing the requirements on the output electron pulse, employing longer laser wavelengths, and increasing the laser pulse energy (see Fig. 3) are also ways of increasing the amount of charge that can be accelerated. We chose a uniform spheroidal distribution for our initial electron pulse due to the well-behaved nature of such distributions^59^, but simulations with other distributions (e.g., a truncated Gaussian distribution) give practically identical results.

Figure 3 shows the results of the scheme for larger laser pulse energies. For instance, we see that final energies of 61 MeV (0.5% energy spread) and 205 MeV (0.25% energy spread) can be attained with 250 mJ and 2.5 J laser pulses respectively (this is further discussed in SI Sections S3 and S4). Further simulations reveal that the scaling of the maximum kinetic energy gain Δ*U* obeys Δ*U*∝*P* ^1/2^ (*P* being peak pulse power) for a fixed electromagnetic pulse duration, in agreement with previous predictions about linear-field particle acceleration^39, 40, 45^. This corresponds to a linear scaling in peak electric field, confirming that our accurate multi-particle computational experiment predicts a linear-field acceleration mechanism.

**Figure 4:**
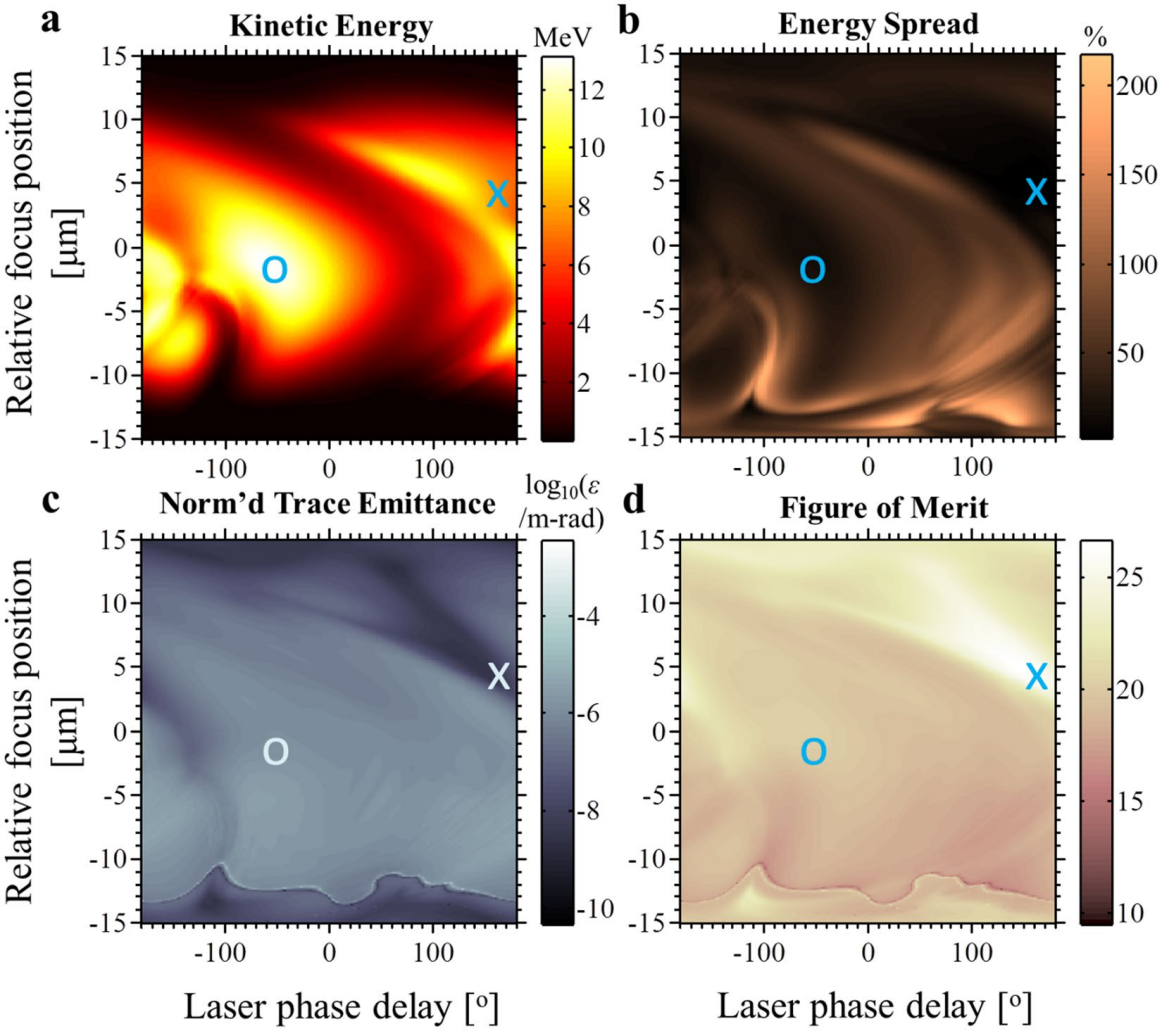
Determining optimal parameters for free space linear-field particle acceleration. The panels show large regimes of high-quality acceleration, allowing optimization of the scheme under different figures-of-merit (FOMs). (**a**) Mean kinetic energy, (**b**) energy spread, (**c**) normalized trace-space emittance and (**d**) FOM of the final electron bunch after acceleration as a function of the optical carrier phase and the position of the electron beam focus. Along the vertical axes, we vary the position of the interaction point (where the electron pulse reaches its focus) relative to the spatial focus of the laser. Along the horizontal axes, we vary the laser carrier phase across all possible phase delays. The laser pulse and initial electron pulse are identical to those used in Figs 1 and 2. In every case, the electron pulse is designed to reach its temporal focus and its spatial focus simultaneously. The temporal focus of the laser pulse is synchronized to coincide with that of the electron pulse. All properties are recorded long after the laser-electron interaction has ceased. The location corresponding to maximum net acceleration is marked with a circle (‘o’), whereas the location of the optimal solution according to our FOM (which takes emittance and energy spread into account; see text) is marked with a cross (‘x’). This optimal solution corresponds to the results in Figs 1 and 2.

Both Figs 3 and 4 emphasize the stability of our scheme by showing that small changes in parameters do not affect the results considerably. For example, a displacement from the optimal point by 1 μm in focal position and 10° in phase delay in Fig. 4 would only degrade the peak acceleration by 1% and the emittance by 5%. Our acceleration scheme is also robust to variations in initial electron energy spread (SI Section S5) and pulse duration (SI Section S6), giving monoenergetic, high-emittance acceleration of multi-electron pulses even with initial electron energy spreads as large as 40% or with a laser pulse duration of 6 fs.

The optimal combination of parameters for the linear-field particle acceleration scheme is ultimately subjective since different applications have different requirements for energy, energy spread and emittance. As Fig. 4 illustrates, the maximum energy gain (marked ‘o’) does not in general correspond to the best normalized trace-space emittances and energy spread. Heuristically, we have found that a useful figure-of-merit (FOM) in determining arguably optimal conditions is ${\rm{F}}{\rm{O}}{\rm{M}}\equiv {(\langle \gamma \rangle -1)}^{5}/({\varepsilon }_{{\rm{x}}}{\varepsilon }_{{\rm{y}}}{\rm{\Delta }}{\gamma }^{4})$, where $\gamma$ is the relativistic Lorentz factor, Δ*γ* the root-mean-square spread in $\gamma$, and ${\varepsilon }_{{\rm{x}},{\rm{y}}}$ the normalized trace-space emittances of the final electron pulse. The parameters used in Figs 1 and 2 were obtained using this FOM (which maximum is marked ‘x’ in Fig. 4). Note that the point of maximum energy gain is not located at the laser focus and zero carrier-envelope phase, since the focus has both the strongest electric field amplitude, which favors large acceleration, as well as the most superluminal phase velocity, which encourages phase slippage and thus hinders acceleration. It is likely that optimizing over even more degrees of freedom can further improve the properties of the accelerated pulse. These degrees of freedom include the optical pulse duration and the spatiotemporal structure of the laser pulse, which one can control with external optical components.

Let us now address an intriguing question: How is it that linear-field acceleration of charged particles in free space has never been observed in laser-driven acceleration experiments, although this is possible over a relatively wide range of parameters? The immediate reason, which also highlights an important requirement in our scheme, is the use of ultrashort pulses that are also of significant intensities (importantly, this requirement falls within the reach of current experimental capabilities). Our findings thus strongly motivate the development of ultra-intense, few-cycle and even sub-cycle laser pulses, as well as better control over the polarization and phase of such pulses. We note that single-cycle pulses^60, 61^, radially-polarized few-cycle pulses^51^ and few-cycle pulses of multi-terawatt peak power^62^ have been experimentally demonstrated.

Our findings shed new light on a fundamental question in light-matter interactions: it is well known that first-order electron-photon interactions in free space (an interaction that involves a single photon, i.e., a single vertex diagram in quantum electrodynamics) are forbidden by energy-momentum conservation laws. This directly implies that any acceleration process in free space – regardless of pulse duration, intensity, or shape – must involve second-order (or higher) electron-photon interactions (interactions that involve more than a single photon, i.e., diagrams containing two or more vertices in quantum electrodynamics)^16, 63^. At the same time, one would expect a higher-order interaction to result in an energy gain that is proportional to a higher power of the field amplitude. For example, ponderomotive acceleration is a second-order effect, and the electron energy gain scales quadratically with the field amplitude even at large field amplitudes^35^. Since first-order interactions are forbidden in free space, we expect a linear scaling of energy gain with field amplitude to be impossible. Our results show, however, that this linear scaling can and does occur for specially-shaped electromagnetic modes when the field amplitude is high enough and the pulse duration short enough. This strongly suggests that the nature of electron-photon interactions starts changing when we leave the realm of perturbative physics, and enter the realm of strong fields and ultrashort pulse interactions. Our results therefore motivate a quantum electrodynamical investigation of electron acceleration in strong and ultrashort fields, where the emergence of ultrastrong coupling and other quantum effects in light-matter interactions may change our basic understanding of electron-photon interactions, even in free-space.

The results described in this work give insights beyond our range of parameters and our choice of laser wavelength, because solutions to the Newton-Lorentz equation of motion and Maxwell’s equations are scale invariant in the absence of space charge. Except for a scaling factor in laser and electron pulse parameters, we expect our results to remain relevant at other wavelengths, modulo some correction terms due to space charge. Terahertz sources have observed a steady trend toward pulses of higher intensity and energy^64–66^, and could be attractive alternatives to optical or infrared sources due to the larger amount of charge that can be accommodated at terahertz wavelengths.

Our results also strongly suggest the viability of linearly accelerating other types of charged particles in free space (e.g., protons and ions, for applications like hadron therapy in cancer treatment and lithography by ion beam milling). Generally, the charged particles may be externally injected, and do not have to be introduced by methods like ionization^67^ that require media or material structures near the laser focus. Weaker inter-particle interactions between heavier charged particles may enable higher current and even more impressive performance of the acceleration scheme. Additionally, note that the linear-field acceleration gradient is given by electric field amplitude *E*, whereas the ponderomotive acceleration gradient is given by $q{E}^{2}/(2\omega \gamma m{\rm{c}})$ ^68^, where *q* and *m* are respectively the particle’s charge and rest mass, $\omega$ is the central angular frequency of the laser, $\gamma$ is the relativistic Lorentz factor, and c is the speed of light in free space. As a result, heavier charged particles (with larger *m*) are likely to be more strongly favored by linear-field acceleration.

In conclusion, we have shown that net energy transfer via linear-field forces, between a laser pulse and a bunch of multiple interacting electrons, can be realized in unbounded free space by engineering the spatiotemporal structure of light. Our findings motivate the development of ultra-intense, few-cycle and even sub-cycle laser pulses, as well as better control over the polarization and phase of such pulses. Rapid technological advances in engineering arbitrary wavefronts and polarizations^69^, together with emerging techniques for precise structuring of electron pulses^70, 71^, create a wealth of opportunities that will push the limits of particle acceleration to ever-higher energies.

## Electronic supplementary material

Laser-Induced Linear-Field Particle Acceleration in Free Space Supplementary Video

Laser-Induced Linear-Field Particle Acceleration in Free Space Supplementary Information

## References

[1] Tajima T, Dawson JM (1979). Laser electron acceleration. Phys. Rev. Lett..

[2] Mourou GA, Tajima T, Bulanov SV (2006). Optics in the relativistic regime. Rev. Mod. Phys..

[3] Salamin YI (2006). Electron acceleration from rest in vacuum by an axicon Gaussian laser beam. Phys Rev A..

[4] Malka V (2008). Principles and applications of compact laser–plasma accelerators. Nat. Phys..

[5] Hafz NAM (2008). Stable generation of GeV-class electron beams from self-guided laser–plasma channels. Nat. Photon..

[6] Norreys PA (2009). Laser-driven particle acceleration. Nat. Photon..

[7] Esarey E, Schroeder CB, Leemans WP (2009). Physics of laser-driven plasma-based electron accelerators. Rev Mod Phys..

[8] Di Piazza A, Müller C, Hatsagortsyan KZ, Keitel CH (2012). Extremely high-intensity laser interactions with fundamental quantum systems. Rev. Mod. Phys..

[9] Peralta EA (2013). Demonstration of electron acceleration in a laser-driven dielectric microstructure. Nature.

[10] Hooker SM (2013). Developments in laser-driven plasma accelerators. Nat. Photon..

[11] Mourou G, Brocklesby B, Tajima T, Limpert J (2013). The future is fibre accelerators. Nat. Photon..

[12] England RJ (2014). Dielectric laser accelerators. Rev. Mod. Phys..

[13] Lawson JD (1979). Lasers and accelerators. IEEE Transactions on Nuclear Science NS.

[14] Woodward PM (1947). A method of calculating the field over a plane aperture required to produce a given polar diagram. J.I.E.E..

[15] Palmer RB (1980). A laser-driven grating linac. Particle Accelerators.

[16] Palmer RB (1995). Acceleration theorems, AIP Conference Proceedings.

[17] Breuer J, Hommelhoff P (2013). Laser-based acceleration of nonrelativistic electrons at a dielectric structure. Phys. Rev. Lett..

[18] Plettner T (2005). Visible-laser acceleration of relativistic electrons in a semi-infinite vacuum. Phys Rev Lett..

[19] Sprangle P, Esarey E, Krall J, Ting A (1996). Vacuum laser acceleration. Opt. Communications.

[20] Marceau V, Varin C, Piché M (2013). Validity of the paraxial approximation for electron acceleration with radially polarized laser beams. Opt. Lett..

[21] Strickland D, Mourou G (1985). Compression of amplified chirped optical pulses. Opt. Communication.

[22] Modena A (1995). Electron acceleration from the breaking of relativistic plasma waves. Nature.

[23] Mangles SPD (2004). Monoenergetic beams of relativistic electrons from intense laser–plasma interactions. Nature.

[24] Geddes CGR (2004). High-quality electron beams from a laser wakefield accelerator using plasma-channel guiding. Nature.

[25] Faure J (2004). A laser–plasma accelerator producing monoenergetic electron beams. Nature.

[26] Wang WT (2016). high-brightness high-energy electron beams from a laser wakefield accelerator via energy chirp control. Phys. Rev. Lett..

[27] Schmid K (2009). Few-Cycle Laser-Driven Electron Acceleration. Phys. Rev. Lett..

[28] Guenot D (2017). Relativistic electron beams driven by kHz single-cycle light pulses. Nat. Photon..

[29] Kimura WD (1995). Laser acceleration of relativistic electrons using the inverse Cherenkov effect. Phys. Rev. Lett..

[30] Kimura WD (2001). First Staging of Two Laser Accelerators. Phys. Rev. Lett..

[31] Duris J (2014). High-quality electron beams from a helical inverse free-electron laser accelerator. Nat. Communications.

[32] Schächter L (1995). PASER: Particle acceleration by stimulated emission of radiation. Phys. Lett. A.

[33] Malka G, Lefebvre E, Miquel JL (1997). Experimental observation of electrons accelerated in vacuum to relativistic energies by a high-intensity laser. Phys. Rev. Lett..

[34] Thévenet M (2016). Vacuum laser acceleration of relativistic electrons using plasma mirror injectors. Nat. Phys..

[35] Hartemann FV (1995). Nonlinear ponderomotive scattering of relativistic electrons by an intense laser field at focus. Phys. Rev. E.

[36] Jackson, J. D. *Classical Electrodynamics* (John Wiley & Sons, New York) 3rd ed (1999).

[37] Marceau V, Varin C, Brabec T, Piché M (2013). Femtosecond 240-keV electron pulses from direct laser acceleration in a low-density gas. Phys. Rev. Lett..

[38] Karmakar A, Pukhov A (2007). Collimated attosecond GeV electron bunches from ionization of high-Z material by radially polarized ultra-relativistic laser pulses. Laser Part. Beams.

[39] Fortin P-L, Piché M, Varin C (2010). Direct-field electron acceleration with ultrafast radially-polarized laser beams: Scaling laws and optimization. J. Phys. B.

[40] Marceau V, April A, Piché M (2012). Electron acceleration driven by ultrashort and nonparaxial radially polarized laser pulses. Opt. Lett..

[41] Marceau V, Hogan-Lamarre P, Brabec T, Piché M, Varin C (2015). Tunable high-repetition-rate femtosecond few-hundred-keV electron source. J. Phys. B.

[42] Sell A, Kärtner FX (2013). Attosecond electron bunches accelerated and compressed by radially polarized laser pulses and soft-x-ray pulses from optical undulators. J. Phys. B..

[43] Haaland CM (1995). Laser electron acceleration in vacuum. Opt. Communications.

[44] Salamin YI, Mocken GR, Keitel CH (2003). Relativistic electron dynamics in intense crossed laser beams: Acceleration and Compton harmonics. Phys. Rev. E.

[45] Wong LJ, Kärtner FX (2010). Direct acceleration of an electron in infinite vacuum by a pulsed radially-polarized laser beam. Opt Express.

[46] Wong LJ, Kärtner FX (2011). A threshold for laser-driven linear particle acceleration in unbounded vacuum. Appl. Phys. Lett..

[47] Gupta DN, Kant N, Kim DE, Suk H (2007). Electron acceleration to GeV energy by a radially polarized laser. Phys. Lett. A.

[48] Pang J (2002). Subluminous phase velocity of a focused laser beam and vacuum laser acceleration. Phys. Rev. E.

[49] Popov KI, Bychenkov V, Yu., Rozmus W, Sydora RD (2008). Electron vacuum acceleration by a tightly focused laser pulse. Phys. Plasma.

[50] Dorn R, Quabis S, Leuchs G (2003). Sharper focus for a radially polarized light beam. Phys. Rev. Lett..

[51] Carbajo S (2014). Efficient generation of ultra-intense few-cycle radially polarized laser pulses. Opt. Lett..

[52] April A, Piché M (2010). 4π Focusing of TM01 beams under nonparaxial conditions. Opt. Express.

[53] Bahk SW (2004). Generation and characterization of the highest laser intensities (10^22^ W/cm2. Opt. Lett..

[54] Floettmann K (2003). Some basic features of the beam emittance. Phys. Rev. Spec. Top.-Ac..

[55] Wong LJ, Freelon B, Rohwer T, Gedik N, Johnson SG (2015). All-optical, three-dimensional electron pulse compression. New J. Phys..

[56] Hilbert SA, Uiterwaal C, Barwick B, Batelaan H, Zewail AH (2009). Temporal lenses for attosecond and femtosecond electron pulses. P. Natl. Acad. Sci. USA.

[57] Hommelhoff P, Sortais Y, Aghajani-Talesh A, Kasevich MA (2006). Field emission tip as a nanometer source of free electron femtosecond pulses. Phys. Rev. Lett..

[58] Hoffrogge J (2014). Tip-based source of femtosecond electron pulses at 30 keV. J. Appl. Phys..

[59] Luiten OJ, van der Geer SB, de Loos MJ, Kiewiet FB, van der Wiel MJ (2004). How to realize uniform three-dimensional ellipsoidal electron bunches. Phys. Rev. Lett..

[60] Huang S-W (2011). High-energy pulse synthesis with sub-cycle waveform control for strong-field physics. Nat. Photon..

[61] Rivas DE (2017). Next Generation Driver for Attosecond and Laser-plasma Physics. Sci. Rep..

[62] Manzoni C (2015). Coherent pulse synthesis: towards sub-cycle optical waveforms. Laser & Photonics Reviews.

[63] Schächter, L. *Beam-Wave in Periodic and Quasi-Periodic Structures* (Springer-Verlag Berlin Heidelberg) 2^nd^ ed., pgs. 2–4 (2011).

[64] Haberberger D, Tochitsky S, Joshi C (2010). Fifteen terawatt picosecond CO_2_ laser system. Opt. Express.

[65] Hoffmann MC, Fülöp JA (2011). Intense ultrashort terahertz pulses: generation and applications. J. Phys. D.

[66] Huang S-W (2013). High conversion efficiency, high energy terahertz pulses by optical rectification in cryogenically cooled lithium niobate. Opt. Lett..

[67] Salamin YI, Harman Z, Keitel CH (2008). Direct high-power laser acceleration of ions for medical applications. Phys. Rev. Lett..

[68] Esarey E, Sprangle P, Krall J (1995). Laser acceleration of electrons in vacuum. Phys. Rev. E.

[69] Sato M (2013). Terahertz polarization pulse shaping with arbitrary field control. Nat. Photon..

[70] Harris J (2015). Structured quantum waves. Nat. Phys..

[71] Feist A (2015). Quantum coherent optical phase modulation in an ultrafast transmission electron microscope. Nature.

